# Critical role for arginase 2 in obesity-associated pancreatic cancer

**DOI:** 10.1038/s41467-017-00331-y

**Published:** 2017-08-14

**Authors:** Tamara Zaytouni, Pei-Yun Tsai, Daniel S. Hitchcock, Cory D. DuBois, Elizaveta Freinkman, Lin Lin, Vicente Morales-Oyarvide, Patrick J. Lenehan, Brian M. Wolpin, Mari Mino-Kenudson, Eduardo M. Torres, Nicholas Stylopoulos, Clary B. Clish, Nada Y. Kalaany

**Affiliations:** 1 Division of Endocrinology, Center for Basic and Translational Obesity Research, Boston Children’s Hospital, Boston, MA 02115 USA; 2 Department of Pediatrics, Harvard Medical School, Boston, MA 02115 USA; 3 Broad Institute of Harvard and MIT, Cambridge, MA 02142 USA; 4 Whitehead Institute for Biomedical Research, Cambridge, MA 02142 USA; 5 Department of Medical Oncology, Dana-Farber Cancer Institute and Department of Medicine, Harvard Medical School, Boston, MA 02215 USA; 6 Department of Pathology, Massachusetts General Hospital and Harvard Medical School, Boston, MA 02114 USA; 7 Department of Molecular, Cell and Cancer Biology, University of Massachusetts Medical School, Worcester, MA 01605 USA; 8 Metabolon Inc, Research Triangle Park, Durham, NC 27709 USA

## Abstract

Obesity is an established risk factor for pancreatic ductal adenocarcinoma (PDA). Despite recent identification of metabolic alterations in this lethal malignancy, the metabolic dependencies of obesity-associated PDA remain unknown. Here we show that obesity-driven PDA exhibits accelerated growth and a striking transcriptional enrichment for pathways regulating nitrogen metabolism. We find that the mitochondrial form of arginase (ARG2), which hydrolyzes arginine into ornithine and urea, is induced upon obesity, and silencing or loss of *ARG2* markedly suppresses PDA. In vivo infusion of ^15^N-glutamine in obese mouse models of PDA demonstrates enhanced nitrogen flux into the urea cycle and infusion of ^15^N-arginine shows that *Arg2* loss causes significant ammonia accumulation that results from the shunting of arginine catabolism into alternative nitrogen repositories. Furthermore, analysis of PDA patient tumors indicates that ARG2 levels correlate with body mass index (BMI). The specific dependency of PDA on ARG2 rather than the principal hepatic enzyme ARG1 opens a therapeutic window for obesity-associated pancreatic cancer.

## Abstract (web summary)

Obesity is an established risk factor for pancreatic ductal adenocarcinoma (PDA). Here the authors show that obesity induces the expression of the mitochondrial form of arginase *ARG2* in PDA and that *ARG2* silencing or loss results in ammonia accumulation and suppression of obesity-driven PDA tumor growth.

## Introduction

Pancreatic ductal adenocarcinoma (PDA) is a deadly malignancy with an incidence on the rise^1, 2^. Parallel to this rise has been a surge in obesity and the metabolic syndrome, established risk factors for PDA^3–7^. Recent studies of tumor metabolism identified *KRAS*-driven alterations in nutrient scavenging and utilization that are critical for the maintenance of PDA tumors^8–10^, the vast majority of which (>90%) harbor activating *KRAS* mutations. However, whether these or other metabolic alterations occur in obesity-associated PDA remains unknown. Here we perform unbiased transcriptomic and metabolomic analyses on orthotopic transplant models of PDA, and identify striking alterations in nitrogen metabolism in PDA tumors of the obese. In particular, we find that arginase 2 (ARG2), the extrahepatic mitochondrial enzyme that catabolizes arginine into ornithine and urea is induced upon obesity. This induction is accompanied by enhanced PDA growth and increased nitrogen flux from ^15^N-glutamine into the urea cycle, the principal mammalian pathway for ammonia detoxification, usually operating in the liver. We find that silencing or loss of *ARG2* in human or mouse tumors respectively, strongly suppresses PDA growth, particularly in obese hosts. Infusion of ^15^N-arginine in murine orthotopic transplant models of PDA demonstrates that *Arg2* deficiency causes shunting of arginine catabolism away from the urea cycle and into creatine synthesis, resulting in significant accumulation of ammonia specifically in tumors of the obese. Our findings point to an essential in vivo role for ARG2 in pancreatic cancer, an insidious malignancy with enhanced protein breakdown and remodeling^11–16^, and suggest a potential need for the channeling of nitrogen into the urea cycle in highly proliferative tumors. Importantly, because ARG1 and not ARG2 is the main hepatic ureagenic enzyme in mammals^17–21^, specific targeting of ARG2 may provide a therapeutic opportunity for the treatment of pancreatic cancer patients, particularly those suffering from obesity and the metabolic syndrome.

## Results

### Nitrogen metabolism genes are induced in PDA of the obese

**Fig. 1:**
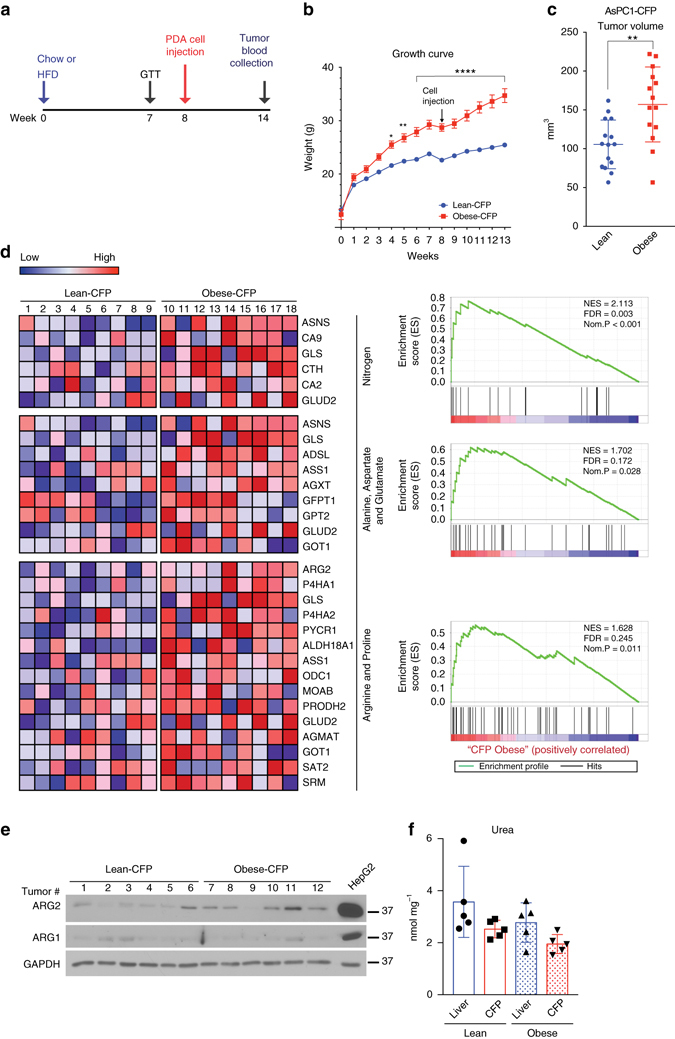
Obesity leads to enhanced PDA growth and transcriptional induction of nitrogen and arginine metabolism. **a** Schematic timeline for the generation of lean or obese orthotopic xenograft models of PDA, using 4–6 week old male *Rag1* ^−/−^ mice. **b** Growth curves of Lean-CFP (*n* *=* 15) or Obese-CFP (*n* *=* 14) mice. *Arrow* indicates orthotopic injection of AsPC1-CFP cells (10^5^ per mouse). **c** Volumes of orthotopic PDA tumors from mice in **b**. **d** Heatmaps (*left*) of genes enriched in metabolic pathways in Lean-CFP compared to Obese-CFP tumors (*n* = 9 per group), with corresponding GSEA plots (*right*). *Red* indicates *higher expression*, and *blue lower expression*, relative to the mean expression level within each group. NES normalized enrichment scores, FDR false discovery rate, *Nom.* nominal. **e** Immunoblots for ARG2 and ARG1 in representative orthotopic tumors from **c**. **f** Graph demonstrating that the urea levels in PDA tumors are comparable to those in the liver of lean or obese mice (*n* = 5); data are representative of three independent experiments. In **b**, data represent the mean ± s.e.m. In **c**, **f**, data represent the mean ± s.d. *P ≤ 0.05, **P *≤* 0.01, ****P *≤* 0.0001, two-way ANOVA followed by Tukey test in **b** and *t*-test in **c**

To identify metabolic dependencies in obesity-driven pancreatic cancer, *KRAS*-mutant human AsPC-1 cells expressing cerulean fluorescent protein (AsPC1-CFP) were used to generate lean and obese orthotopic xenograft models of PDA, as outlined in Fig. 1a. High fat diet-fed “obese” mice gained significantly more weight (*P* ≤ 0.0001, two-way ANOVA followed by Tukey test) than their chow-fed “lean” counterparts (Fig. 1b and Supplementary Fig. 1a) and displayed features of the metabolic syndrome (Supplementary Fig. 1b–e). The lean and obese mice were then injected orthotopically into their pancreata with AsPC1-CFP cells and maintained on their corresponding diets for 6 weeks prior to tumor harvest (Fig. 1a). The resulting tumors were termed “Lean-CFP” and “Obese-CFP”, according to the metabolic state of the mouse. Consistent with a role for obesity in promoting PDA growth, Obese-CFP tumors grew significantly larger (1.5-fold, *P* = 0.002, *t*-test) than Lean-CFP (Fig. 1c). A mild stromal infiltration was observed in all PDA tumors, independent of obesity (Supplementary Fig. 2).

To identify obesity-associated transcriptional alterations in PDA, comparative microarray expression analysis was performed. Gene set enrichment analysis (GSEA)^22^ revealed a striking over-representation of KEGG-defined pathways involved in “Nitrogen Metabolism” (FDR *q*-value = 0.003; *P* < 0.001), “Alanine, Aspartate and Glutamate Metabolism” (FDR *q*-value = 0.172; *P* = 0.028), and “Arginine and Proline Metabolism” (FDR *q*-value = 0.245; *P* = 0.011) in Obese-CFP compared to Lean-CFP tumors (Fig. 1d). Notably, there was increased expression of glutaminase (*GLS*) and glutamate dehydrogenase (*GLUD*), which catalyze the deamination of glutamine and glutamate, respectively, generating ammonia. Expression of aspartate transaminase (*GOT*), which transfers the amino group from glutamate to oxaloacetate (OAA), generating aspartate was also upregulated (Fig. 1d). Moreover, there was induction in the rate-limiting enzyme in arginine biosynthesis, argininosuccinate synthetase (*ASS1*). In addition, mRNA levels of the mitochondrial, extrahepatic form of arginase (*ARG2*) were elevated. This is in contrast to its paralog (*ARG1*), which codes for the hepatic cytosolic form of arginase, the principal ureagenic enzyme that catalyzes the final step in nitrogen excretion and ammonia detoxification (Fig. 1d). Whereas ARG1 protein levels were faintly detected, those of ARG2 were induced in Obese-CFP compared to Lean-CFP tumors (Fig. 1e). These results imply a potential role for the urea cycle in PDA growth, as nitrogens from both ammonia and aspartate get incorporated into arginine and ultimately urea. Importantly, urea levels in the tumors were comparable to those in the liver, where the urea cycle largely operates, indicating that this pathway is indeed active in PDA (Fig. 1f).

### ARG2 is critical for PDA growth particularly in the obese

**Fig. 2:**
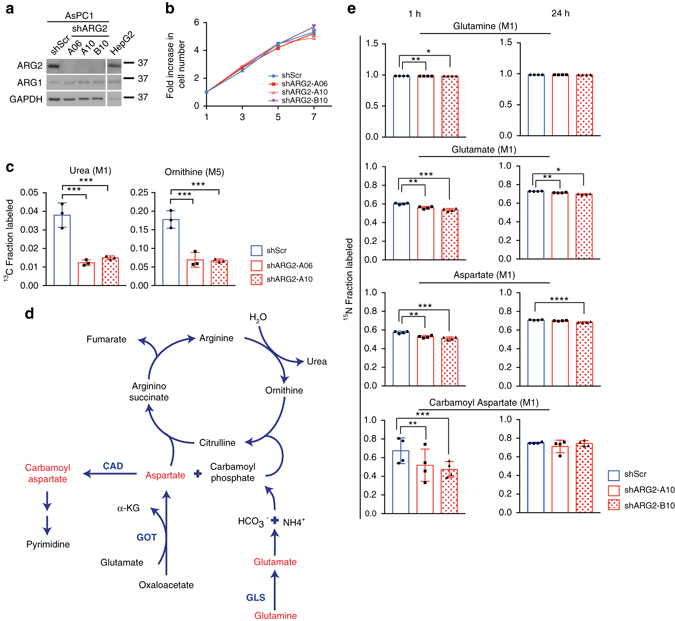
Glutamine nitrogens do not integrate into the urea cycle in vitro. **a** Immunoblots for ARG2 and ARG1 in AsPC1-shScr and AspC1-shARG2 cells. **b** Proliferation curves of cells in **a** (*n* = 6). Data are representative of 4 independent experiments. **c** Fractional ^13^C-labeling of urea (M1) and ornithine (M5) in AsPC-1 cells with control (shScr) or *ARG2* knockdown (shARG2, hairpins A06 and A10) grown in culture in 0.1% serum and 100 ng ml^−1^ IGF-1, supplemented with 1 mM ^13^C_6_-arginine for 48 h (*n* = 4). **d** Diagram of the urea cycle, with metabolites in red indicating significant ^15^N-labeling. **e** Fractional labeling of the indicated metabolites in cells described in **a**, grown in media supplemented with 2 mM ^15^N(amine)-glutamine for 1 h or 24 h (n = 4). Data are representative of two independent experiments. In **b**, data represent the mean ± s.e.m. In **c**, **e**, data represent the mean ± s.d. *P ≤ 0.05, **P ≤ 0.01, ***P ≤ 0.001, ****P ≤ 0.0001, by one-way ANOVA followed by Tukey test

To investigate its role in PDA tumor growth, *ARG2* was knocked-down in AsPC-1 cells (Fig. 2a). Compared with control scramble knockdown (AsPC1-shScr), *ARG2* silencing (AsPC1-shARG2) did not significantly affect PDA cell proliferation in vitro (Fig. 2b), nor did it lead to compensatory increases in ARG1 levels (Fig. 2a). This was consistent among other human PDA cells (Supplementary Fig. 3). To assess ARG2 activity, AsPC1-shScr and AsPC1-shARG2 cells were labeled with ^13^C_6_-arginine, followed by a measure of ^13^C-labeled ARG2 reaction products by liquid chromatography-mass spectrometry (LC–MS). Enrichment in ^13^C-labeled urea M1 (3.8%) and ornithine M5 (17.8%) was noted in the AsPC1-shScr control cells. These levels were strongly suppressed (urea 1.4%; ornithine 6.8%) upon *ARG2* silencing (*P* ≤ 0.001, one-way ANOVA followed by Tukey test, Fig. 2c), indicating that ARG2 was indeed active in PDA cells. Surprisingly however, when the cells were labeled with ^15^N(amine)-glutamine, ^15^N-labeling was detected in glutamate, aspartate and carbamoyl aspartate (60–70%), the precursor of pyrimidine synthesis (Fig. 2d, e), but not in urea cycle metabolites citrulline, arginine or urea. These results indicate that although ARG2 is expressed and active in PDA cells in vitro, the amine nitrogen of glutamine is not disposed of through the urea cycle.

**Fig. 3:**
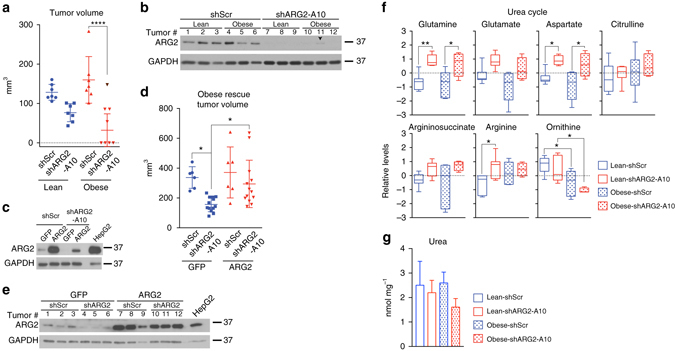
*ARG2* is essential for human PDA growth particularly in the obese. **a** Volumes of orthotopic PDA tumors derived from AsPC1-shScr and AspC1-shARG2 cells, injected (10^5^ cells) into lean or obese 12–14 week old male *Rag1* ^−/−^ mice and grown for 6 weeks (*n* = 7 except for obese-shARG2, *n* = 8). The *black data point* indicates an outlier AspC1-shARG2 tumor not included in the statistics, due to re-expression of ARG2. **b** Immunoblots for ARG2 in tumors from **a**. *Arrowhead* indicates outlier in **a**. **c** Immunoblots for ARG2 in AsPC1-shScr and AsPC1-shARG2 cells expressing GFP or shRNA-resistant human ARG2. **d** Volumes of orthotopic PDA tumors from cells in **c**, grown in obese 27-week old male *Rag1* ^−/−^ mice for 4 weeks (500,000 cells per mouse, *n* = 6 for shScr groups and *n* = 13 for shARG2 groups). Data represent two independent experiments. **e** Immunoblots for ARG2 from tumors in **d**. **f** Relative metabolite levels in PDA tumors from **a** (*n* = 7, except for Obese-shARG2, *n* = 4). *Box plots* represent medians ± 10-90 percentile. **g** Urea levels in tumors from **f** measured by urea assay kit. In **a**, **d**, **g**, data indicate the mean ± s.d. *P ≤ 0.05, **P ≤ 0.01, ****P ≤ 0.0001, by one-way ANOVA followed by Tukey test in **a**, **d**, **f**

Because ARG2 was induced in obesity-associated tumors (Fig. 1d, e), we asked whether its role in PDA growth was relevant in an in vivo, rather than in vitro setting. When PDA cells were grown as orthotopic xenografts in lean or obese mice (Fig. 1a and Supplementary Fig. 4a, b), a moderate decrease in tumor volume was noted in lean mice upon *ARG2* knockdown (1.7-fold, Fig. 3a). However, this effect was more dramatic in obese mice, where only half of the AsPC1-shARG2 tumors grew to a measurable size, reaching volumes 2.1-fold smaller than those of the control shScr tumors (*P* ≤ 0.0001, one-way ANOVA followed by Tukey test), with the exception of one AsPC1-shARG2 tumor that was similar in size to the control knockdown (~150 mm^3^, black data point, Fig. 3a). Consistent with a strong dependence of obesity-associated PDA on ARG2, this tumor had re-expressed ARG2 in vivo (Fig. 3b, lane 11). Introduction of a short hairpin RNA-resistant form of human ARG2 (Fig. 3c) rescued the growth of these tumors compared to tumors overexressing control GFP, where *ARG2* knockdown caused a 2.2-fold suppression (*P* ≤ 0.05, one-way ANOVA followed by Tukey test, Fig. 3d, e).

To investigate the role of ARG2 in the metabolism of pancreatic tumors, we performed unbiased global metabolite profiling and found that nitrogen-centric pathways enriched in urea cycle metabolites were highly impacted by *ARG2* knockdown (Supplementary Fig. 4c). Indeed, *ARG2* suppression led to a block in the urea cycle in tumors from both lean and obese animals, causing significant accumulation of glutamine, aspartate, arginine, a less significant but evident increase in the levels of glutamate, citrulline and argininosuccinate, and a concomitant decrease in ornithine levels (*P* ≤ 0.05, one-way ANOVA followed by Tukey test, Fig. 3f). Moreover, although urea is a highly soluble, secreted metabolite, a modest decrease in its levels was nonetheless observed in tumors of the obese but not the lean (Fig. 3g). Altogether, these data suggest an increased dependence of obesity-associated tumors on ARG2 for nitrogen disposal.

### Obesity enhances nitrogen flux into the urea cycle in PDA

**Fig. 4:**
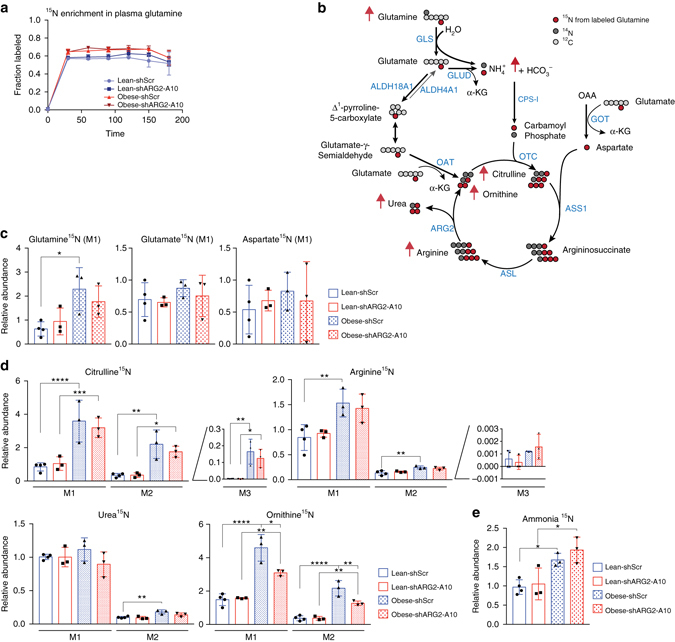
PDA tumors of the obese have enhanced nitrogen flux into the urea cycle. **a** ^15^N enrichment in plasma glutamine in 19–21 week old male *Rag1* ^−/−^ mice bearing orthotopic AsPC1-shScr and AspC1-shARG2 PDA tumors that were infused with ^15^N(amine) glutamine (*n* *=* 3). **b** Schematic of the urea cycle with *red arrows* indicating increases in ^15^N-labeling of the urea cycle metabolites in tumors of the obese; CPS-1, Carbamoyl phosphate synthase 1; OTC, Ornithine transcarbamylase; ASL, Argininosuccinate lyase. **c**, **d** Relative abundance of ^15^N-labeled metabolites (*n* = 3 except for Lean-shScr *n* = 4) outside **c**, or within **d** the urea cycle. **e** Relative abundance of derivatized ^15^N-ammonia. Data indicate the mean ± s.d. in **a**, **c**–**e**, and are representative of two independent experiments. *P ≤ 0.05, **P ≤ 0.01, ***P *≤* 0.001, ****P ≤ 0.0001, by one-way ANOVA followed by Tukey test

To trace the fate of nitrogen from glutamine into the urea cycle, lean, and obese mice bearing orthotopic PDA tumors were infused over a period of 3 h with ^15^N(amine)-glutamine, reaching steady-state plasma enrichment levels of ~60% within the first 30 min (Fig. 4a, b). This enrichment led to the ^15^N-labeling of glutamine, glutamate, and aspartate in PDA tumors (Fig. 4c). However, in contrast to the results obtained from cultured PDA cells (Fig. 2d, e), ^15^N-labeling was also detected in citrulline M1, arginine M1 and M2, and urea M1 and M2, reaching significantly higher levels in tumors of the obese compared with the lean (*P* ≤ 0.01, one-way ANOVA followed by Tukey test, Fig. 4d). These results provide evidence for an in vivo nitrogen flow from glutamine into urea in PDA tumors, and indicate that this flow is further enhanced in an obese state.

Interestingly, although arginine donates its glutamine-derived nitrogens to urea, but not ornithine, upon arginase activity (Fig. 4b), significant ^15^N-labeling in ornithine M1 and M2 was detected in the PDA tumors (Fig. 4d). This labeling is a result of *de novo* ornithine biosynthesis, catalyzed by ornithine aminotransferase (OAT), where glutamate-dependent transamination of glutamate-γ-semialdehyde, produces ornithine M1 or M2 (Fig. 4b). As a consequence, M2- and M3-labeled citrulline, and to a lesser extent M3-labeled arginine, were also detected, and the abundance of M2- and M3-citrulline was significantly increased in PDA of the obese, compared to the lean (*P* ≤ 0.01, one-way ANOVA followed by Tukey test, Fig. 4b, d). Furthermore, *ARG2* knockdown strongly and specifically suppressed ^15^N-ornithine production in PDA tumors of obese, compared with lean mice (M1, *P* ≤ 0.05 and M2, *P* ≤ 0.01, one-way ANOVA followed by Tukey test, Fig. 4d). The enhanced incorporation of ^15^N from glutamine into the urea cycle through ornithine biosynthesis and its suppression upon *ARG2* silencing provides further evidence for the need to dispose of excess nitrogen in tumors of the obese. Interestingly however, only a mild decrease in urea M1 and M2 levels was observed upon *ARG2* knockdown, likely due to the short duration of the infusions and the secretion of this highly soluble metabolite (Fig. 4d). Because the urea cycle mainly functions to prevent accumulation of toxic ammonia by converting excess nitrogen into ornithine and urea, we quantified ^15^N-ammonia levels derived from ^15^N-glutamine, following its chemical derivatization with dansyl chloride^23^. Consistent with an increase in nitrogen levels in obesity-associated PDA, we found a significant accumulation of ^15^N-ammonia in tumors of the obese, the levels of which tended to further increase upon *ARG2* knockdown (*P* ≤ 0.05, one-way ANOVA followed by Tukey test, Fig. 4e).

### *Arg2* loss causes ammonia accumulation in PDA of the obese

**Fig. 5:**
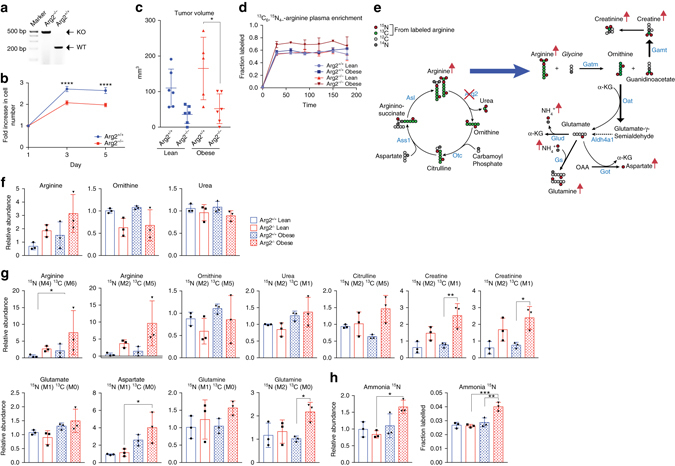
*Arg2* deficiency suppresses obesity-associated mouse PDA and results in ammonia accumulation. **a** DNA gel confirming the genotypes of KPC cells with wild-type (WT) expression or knockout (KO) of *Arg2*. **b** Proliferation curves of cells in **a** (*n* = 6). Data are representative of three independent experiments. **c** Volumes of orthotopic PDA tumors from cells in **b** injected (250,000 per mouse) in lean or obese 11–13 week old male C57BL/6J-129 svJae mice and grown for 2 weeks (*n* = 6 except for Obese-Arg2^+/+^ *n* = 5). **d** ^15^N_4_,^13^C_6_ enrichment in plasma arginine in mice from **c** infused with ^15^N_4_,^13^C_6_-arginine (*n* = 3). **e**, Schematic showing that *Arg2* knockdown in obese PDA causes shunting of arginine away from the urea cycle and towards creatine biosynthesis, along with transfer of nitrogen into glutamate, aspartate, glutamine, and ammonia in *Arg2* ^−/−^ KPC tumors of obese mice. *Red arrows* indicate increases in ^15^N-labeling, of indicated metabolites in *Arg2* ^−/−^ tumors of obese mice. Gamt, Guanidinoacetate N-Methyltransferase. **f** Relative abundance of total pool of the indicated metabolites from tumors in **c**. **g** Relative abundance of the indicated labeled isotopomers in tumors from **c**. **h** Relative abundance (*left*) and fractional labeling (*right*) of derivatized ^15^N-ammonia. Data represent the mean ± s.e.m. in **b**, or mean ± s.d. in **c**, **d**, **f**–**h**. * *P* ≤ 0.05, **P ≤ 0.01 and *** *P* ≤ 0.001, **** *P* ≤ 0.0001, by two-way ANOVA in **b**, and one-way ANOVA in **c**, **f**–**h**, followed by Tukey test

To reinforce our findings and confirm a role for the urea cycle in PDA tumor growth, we sought to generate a PDA model with complete loss, rather than knockdown, of arginase 2. To that end, we bred the genetically engineered mouse model of PDA, *LSL-Kras* ^*G12D*^ *;p53* ^*fl/fl*^; *pdx-1-Cre;* (KPC mice) to *Arg2*-deficient mice, which express an aberrant, inactive form of the protein^21^ (Supplementary Fig. 5a). Resultant tumors were used to generate KPC tumor cell lines with wild-type *Arg2* expression (*Arg2* ^*+/+*^) or *Arg2* loss (*Arg2* ^−/−^) as confirmed by genotyping^21^ (Fig. 5a). *Arg2* loss caused a moderate but significant decrease in the proliferation of KPC cells in vitro (*P* ≤ 0.0001, one-way ANOVA followed by Tukey test, Fig. 5b). These cells were then grown as orthotopic PDA allografts in *Kras-* and *Arg2*-wild-type littermates. Consistent with the results from human PDA (Fig. 1c), mouse PDA tumor growth was accelerated by obesity (Fig. 5c). Importantly, when tumors were harvested at an early stage of progression (average size~ 165 mm^3^), loss of *Arg2* led to a more significant suppression of tumor growth in obese compared to lean mice (3.2-fold, *P* *≤* 0.05, one-way ANOVA, followed by Tukey test, Fig. 5c). This differential effect was strikingly more evident in more advanced tumors (~800 mm^3^), where a dramatic decrease in growth was observed in the obese (5.3-fold, *P* ≤ 0.01, one-way ANOVA, followed by Tukey test), but not in the lean (Supplementary Fig. 5b).

We then investigated the metabolic effect of *Arg2* deficiency on nitrogen metabolism in KPC tumors. We reasoned that infusing heavy labeled arginine, the substrate of Arg2, would convey direct information concerning rapid accumulation of ammonia and alternative fates of excess nitrogen due to *Arg2* loss. To that end, ^15^N_4_,^13^C_6_-labeled arginine was infused for 3 h in lean and obese mice bearing tumors with or without *Arg2* loss (KPC; *Arg2* ^−/−^ or KPC; *Arg2* ^*+/+*^), and metabolic tracing of ^15^N and ^13^C into the urea cycle was performed (Fig. 5d–h). As expected, *Arg2* deficiency led to mild non-significant increases in total levels of arginine and lower levels of ornithine (Fig. 5f). No significant changes in the relative abundance of intratumoral ^15^N-urea M2 were observed in *Arg2* ^−/−^ tumors (Fig. 5g), likely due to the short infusion period, and the secretion of urea from surrounding *Arg2* ^*+/+*^ cells. Nevertheless, a mild decrease in total levels of urea was noted in the *Arg2* ^−/−^ tumors of the obese (Fig. 5f). Consistently, *Arg2* loss resulted in significantly increased levels of ^15^N-arginine M4 and M2 in obesity-associated tumors (*P* ≤ 0.05, one-way ANOVA, followed by Tukey test). This was accompanied by a notable shift in arginine catabolism towards creatine biosynthesis (Fig. 5e, g). The first step in this pathway is catalyzed by Gatm (glycine amidinotransferase, mitochondrial) and involves the transfer of a guanidino group from arginine to glycine, yielding ornithine and guanidinoacetate, the immediate precursor of creatine (Fig. 5e). Indeed, the relative abundance of ^15^N,^13^C-creatine (M2, M1) and creatinine (M2, M1) was significantly increased in tumors with *Arg2* deficiency, particularly in the obese (*P* ≤ 0.05 and *P* ≤ 0.01, respectively, one-way ANOVA followed by Tukey test, Fig. 5g).

Notably, *Arg2* deficiency caused a significant increase in labeled aspartate M1 and glutamine M2, specifically in tumors of the obese (*P* ≤ 0.05, Fig. 5g), accompanied by a striking accumulation in ^15^N-ammonia labeled fraction derived from ^15^N_4_,^13^C_6_-arginine (4% in obese compared to 2.8% in lean, *P* ≤ 0.001, one-way ANOVA followed by Tukey test, Fig. 5h). The specific accumulation of labeled glutamine, aspartate and ammonia from ^15^N_4_,^13^C_6_-arginine in *Arg2*-deficient tumors of the obese results from the shunting of arginine into ornithine by Gatm followed by a transamination reaction catalyzed by Oat to form glutamate-γ-semialdehyde M1 and α-KG-derived glutamate M1 (Fig. 5e). Labeled glutamate can also result from the conversion of Δ^1^-pyrroline-5-carboxylate (P5C, a tautomer of glutamate-γ-semialdehyde, Fig. 4b), by aldehyde dehydrogenase family 4 member A1 (Aldh4a1, Fig. 5e). Excess nitrogen can be transferred from glutamate to aspartate (by Got) or to ammonia (via Glud), while excess ammonia is subsequently utilized by glutamine synthetase (Gs) to generate glutamine (Fig. 5e, g, h). The shunting of arginine from the urea cycle towards alternative nitrogen-containing metabolites (creatine, creatinine, aspartate, and glutamine) upon *Arg2* loss, along with the dramatic accumulation of ammonia specifically in tumors of the obese, emphasize a role for the urea cycle in nitrogen disposal in obesity-associated PDA.

### Enhanced in vivo tumor growth induces ARG2 expression

The crucial role of ARG2 in PDA tumor growth in vivo, but not in vitro, led us to investigate whether candidate metabolites or growth factors that are elevated in obesity^24^ (Supplementary Fig. 1c–e), could lead to the induction of ARG2 expression. Interestingly, treatment of cultured human PDA cells (AsPC-1, HPAC and SUIT-2) with insulin (5–50 ng ml^−1^), insulin-like growth factor-1 (IGF-1, 5–100 ng ml^−1^), glucose (3–11 mM), or a chemically defined lipid mixture (0.1–2%) did not affect ARG2 levels (Supplementary Fig. 6a, b). These results, along with a recognized association of PDA tumors with enhanced protein catabolism^8, 11–15, 25^, led us to hypothesize that obesity-induced PDA tumor growth could generate an in vivo dependency on the urea cycle for disposal of excess nitrogen.

**Fig. 6:**
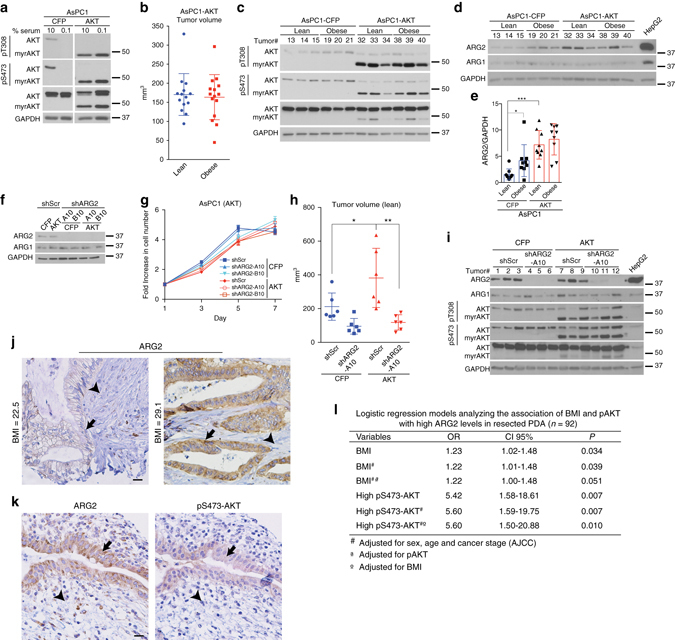
AKT activation enhances in vivo PDA growth and sensitizes tumors to *ARG2* knockdown. **a** Immunoblots for pT308-AKT, pS473-AKT and total AKT in isogenic AsPC1-CFP and AsPC1-AKT PDA cells expressing SRC myristoylation sequence-AKT fusion (myrAKT). **b** Volumes of orthotopic PDA tumors from cells in **a** (10^5^ per mouse) grown for 6 weeks in lean (*n* = 14) or obese (*n* = 15) 12–14 week old male *Rag1* ^−/−^ mice following the outline in (Fig. 1a). **c**, **d** Immunoblots for pT308-AKT, pS473-AKT, and total AKT **c**, ARG2 and ARG1 **d** in tumors from **b**. **e** ARG2 levels as quantified by ImageJ and normalized to GAPDH, from immunoblots in **d**, Fig. 1e, and Supplementary Fig. 10c. **f** Immunoblots of ARG2 and ARG1 in isogenic cells from **a**, harboring control Scramble (shScr) or *ARG2* (shARG2) knockdown. **g** Proliferation curves of cells in **f** (*n* = 6). Data are representative of four independent experiments. **h** Volumes of orthotopic PDA xenografts from cells in **f** that were injected in lean 8–9 week old male *Rag1* ^−/−^ mice (500,000 per mouse) and grown for 5 weeks (*n* = 6). **i** Immunoblots for ARG2, ARG1, pT308-AKT, pS473-AKT and total AKT from representative tumors in **h**. **j**, **k** Representative immunohistochemistry staining showing enhanced ARG2 levels in PDA tumors from patients with higher compared to lower BMI **j**, and overlapping pS473-AKT and ARG2 staining in sequential tumor sections from PDA patients **k**. *Arrows* indicate areas of ductal adenocarcinoma and *arrowheads* indicate stroma. *Scale bars*: 20 µm. **l** Multivariate regression analysis of the association of BMI and pAKT with high ARG2 levels in resected PDA immunostained in **j**, **k**. OR odds ratio, CI confidence interval, AJCC American Joint Committee on Cancer. Data represent the mean ± s.d. in **b**, **e**, **h** or mean ± s.e.m. in **g**. *P ≤ 0.05, **P ≤ 0.01, ***P ≤ 0.001, one-way ANOVA followed by Tukey test

We therefore sought to investigate the role of ARG2 in an obesity-independent model of PDA with enhanced tumor growth. To that end, we generated AsPC-1 PDA cells with constitutive activation of AKT, a kinase known to promote tumor growth and invasion^26, 27^. Unlike AsPC-1 parental and AsPC1-CFP cells, AsPC1-AKT exhibited AKT activation and a proliferative capacity that are independent of serum, insulin or IGF-1 (Fig. 6a and Supplementary Fig. 7a–c). These cells were used to generate “Lean-AKT” and “Obese-AKT” orthotopic tumors, in parallel to the Lean-CFP and Obese-CFP tumors generated from the isogenic AsPC1-CFP cells (Fig. 1a–c, Fig. 6b and Supplementary Fig. 8a–e). Notably, independent of obesity, the volumes of AsPC1-AKT tumors (Lean-AKT and Obese-AKT) were comparable to those of Obese-CFP tumors and 1.6-fold larger than those of Lean-CFP (~170 versus 105 mm^3^, *P* ≤ 0.01 by one-way ANOVA followed by Tukey test, compare Figs. 6b and 1c). Although this suggests a potential role for AKT in mediating the obesity-driven increase in PDA growth^3, 28–32^, no significant changes in AKT phosphorylation (T308 or S473) were detected in Obese-CFP compared to Lean-CFP tumors (*n* = 12, Fig. 6c and Supplementary Fig. 9). These results indicate that obesity and AKT activation are likely to be independent contributors to enhanced in vivo PDA growth.

Strikingly, the transcriptional signature of AsPC1-AKT tumors originating from either lean or obese mice significantly overlapped with that of Obese-CFP, when compared to Lean-CFP tumors (Supplementary Fig. 8f), suggesting that enhanced in vivo tumor growth is a strong inducer of nitrogen metabolism and the urea cycle. It is also possible that these metabolic changes occurred as a result of acinar tissue disruption during AKT- or obesity-driven pancreatic tumor development. Consistently, ARG2 expression was markedly increased in the tumors harboring constitutively active AKT, independent of obesity (*P* ≤ 0.001, one-way ANOVA followed by Tukey test, Fig. 6d, e and Supplementary Fig. 10a–c). Importantly, this increase was only observed in tumors, but not in AsPC1-AKT cells propagated in culture (Fig. 6f and Supplementary Fig. 10d), further highlighting the in vivo, rather than in vitro relevance of ARG2 in PDA tumor growth. It however remains to be investigated whether AKT activation results in an enhanced in vivo nitrogen flux into the urea cycle similar to that observed in obesity-driven PDA. Although the transcriptional mechanism leading to in vivo ARG2 induction is yet to be identified, these results are consistent with a possible reliance on the urea cycle for the disposal of excess nitrogen in highly proliferative hypovascular tumors. Such environment contrasts the in vitro setting, where cells are standardly cultured in copious amounts of media, which allows for the secretion and dilution of nitrogen-containing metabolites. Indeed, *ARG2* suppression did not affect the growth of AsPC1-AKT cells, in vitro (Fig. 6f, g). However, when grown as orthotopic xenografts in lean immunodeficient mice, PDA tumors with AKT activation were larger than control CFP-expressing tumors (1.8-fold, *P* ≤ 0.05), and displayed an enhanced dependency on ARG2 (Fig. 6h, i). Whereas *ARG2* knockdown led to a non-statistically significant two-fold decrease in the volumes of CFP-expressing tumors, a significantly stronger suppression was noted in tumors with AKT activation (3.3-fold, *P* ≤ 0.01, one-way ANOVA followed by Tukey test, Fig. 6h).

### ARG2 levels in PDA correlate with patient BMI

To extend the relevance of our findings, we immunostained PDA tissue microarrays from 92 patients with resected tumors, for ARG1, ARG2, and phospho-S473 AKT (pAKT). Because ARG2 levels increase in diabetic patients and are mitigated by insulin treatment^33–35^, patients with history of diabetes were excluded from the study. Although PDA patients can lose weight as a result of cachexia, over a third of the patient pool (38%) was reported as overweight or obese at time of resection. Whereas significant ARG2 and pAKT staining was observed in the PDA tumors (Fig. 6j, k), ARG1 was either undetected or weakly expressed. Using multivariable-adjusted logistic regression models, we found a significant positive association between BMI and ARG2 levels (*P* = 0.039, Fig. 6j, l). Notably, adjusting for pAKT levels only mildly attenuated the significance but not the strength of this association (*P* = 0.051, Fig. 6l). In contrast, no significant association was found between BMI and pAKT. However, high pAKT levels were strongly associated with high ARG2 expression, independent of BMI (*P* = 0.010, Fig. 6k, l). These results corroborate our findings in the orthotopic PDA tumor models, where AKT and obesity independently induce ARG2 expression.

## Discussion

Our study highlights a role for the urea cycle in maintaining the growth of pancreatic tumors, particularly in overweight or obese systemic metabolic states. The dependency of PDA tumors on ARG2, which catalyzes the final step of the urea cycle, suggests a need to alleviate an increased nitrogen burden, particularly in an obese host, where tumors exhibit enhanced in vivo growth. Pancreatic cancer is recognized for high-basal levels of autophagy^12, 15^ and macropinocytic uptake of extracellular proteins^11, 13, 16^, both of which can lead to enhanced protein breakdown and remodeling^14^ to maintain tumor growth. We therefore propose that conditions that enhance PDA growth in vivo, such as obesity or AKT activation, might further exaggerate this process, generating excess levels of nitrogen and creating a dependency on ARG2. This dependency is specific to tumors but not cultured cancer cells, which lack the in vivo tumor microenvironment and are grown in a constantly replenished medium, preventing the intracellular accumulation of nitrogen-rich metabolites. Although our findings in orthotopic transplant models remain to be confirmed in an autochthonous mouse model of PDA with *Arg2* loss, they are in line with recent reports highlighting the differential effects of in vitro versus in vivo environments on the growth and metabolism of tumors from different tissues^25, 36–38^. It is noteworthy that human disorders have not been associated with *ARG2* deficiency^17^. However, it remains to be investigated whether defects in its activity cause embryonic lethality. Nevertheless, whole body genetic deletion of *Arg2* in mice does not lead to significant abnormalities, except for a mild increase in circulating levels of arginine^21^. This is in contrast with reported deficiencies in the predominantly hepatic *ARG1*, which lead to robust hyperarginemia, neurological impairment, growth retardation and fatal episodes of hyperammonemia^17–20^. Consequently, ARG2 provides an attractive therapeutic target in PDA patients, particularly those suffering from obesity or those with AKT-driven PDA tumors resulting from activating oncogenic mutations, independent of obesity.

## Methods

### Reagents

Antibodies for western blotting: AKT (#4691; 1:2000), pS473-AKT (#4056; 1:1000), pT308-AKT (#4058; 1:500), PTEN (#9552; 1:1000) were obtained from Cell Signaling Technologies, ARG1 (#ab133543; 1:500), human ARG2 (#ab137069; 1:1000), mouse ARG2 (#bs-11397R; 1:2000) from Bioss Antibodies and GAPDH (#sc-25778; 1:5000) from Santa Cruz. Antibodies for immunofluorescence were from Cell Signaling Technologies (pS473-AKT #4060; 1:50), abcam (α-SMA #ab5694; 1:200), Vector Laboratories (Biotinylated DBA-Lectin #B-1035; 1:250), and Sigma-Aldrich (α-Amlyase #A8273; 1:1000). Human recombinant Insulin (Roche, 11376497001), human recombinant IGF-1 (PeproTech, 100-11), D-Glucose (Sigma-Aldrich, G7021) and a chemically defined lipid mixture (sigma L0288) were used to treat cultured cells.

### Cell culture

Human PDA cell lines were from the American Type Culture Collection or ATCC (AsPC-1, HPAC, MIA PaCa-2, PANC-1), Japanese Collection of Research Bioresources (SUIT-2), or the German Collection of Microorganisms and Cell Cultures (PA-TU-8988T) and were authenticated by STR profiling at ATCC. All cell lines tested negative for mycoplasma using LookOut Mycoplasma PCR Kit (Sigma, MP0035). KPC-*Arg2* ^*+/+*^ and KPC-*Arg2* ^−/−^ mouse PDA cell lines were dissociated from corresponding mouse tumors^39^ in Hanks buffered saline solution (HBSS, Invitrogen; calcium/magnesium free) containing 0.025% trypsin–EDTA (Invitrogen) and 1 mg ml^−1^ collagenase IV (Worthington Biochemicals). Following a 2-h incubation with rotation at 37 °C, the samples were triturated, pelleted, resuspended in HBSS, and filtered through 40-μm cell strainers (BD Falcon). All cells were maintained at 37 °C in a humidified incubator with 5% CO_2_ and were grown in RPMI-1640 (Sigma) supplemented with 10% fetal bovine serum and penicillin-streptomycin (Gibco). For metabolic tracing studies in vitro, the culture medium was optimized to exclude arginase activity in 10% serum, as detected by LC–MS. The cells were instead incubated in media supplemented with 0.1% serum and 100 ng ml^−1^ IGF-1. Unless otherwise indicated, all proliferation assays were also performed on cells cultured in 0.1% serum with 100 ng ml^−1^ IGF-1, using the proliferation kit II (XTT; Roche)^32^.

### Expression plasmids

pBabe-neo-nlsCFP and pBabe-neo-ΔPH-myrAKT1 retroviral vectors were a gift from J. Albeck (UC Davis) who had generated them as follows: For pBabe-neo-nlsCerulean, the sequence of Cerulean (a gift from David Piston^40^) was subcloned into pBabe-neo with an SV40 nuclear localization sequence at the 5′ terminus. For pBabe-neo-myr-AKT1(deltaPH)-HA, the sequence for myr-AKT1(deltaPH)-HA was subcloned into pBabe-neo from pWZLneo-myr-Akt(deltaPH)-ER, a gift from R. Roth^41^. pLJM15-neo-GFP was a gift from D.M. Sabatini (Whitehead Institute). The short hairpin (sh)RNA-resistant human ARG2 expression construct pLJM15-neo-hARG2-shA10R was generated as follows: human ARG2 cDNA (Clone ID: 5179861) sequence from pCMV-SPORT6-ARG2 (GE-Dharmacon MHS6278-202800846) was subcloned into the lentiviral vector pLJM15-neo-polylinker (a gift from Dr. D.M. Sabatini) using the AgeI/XbaI restriction sites. This construct was subsequently modified by site-directed mutagenesis (QuikChange II Site-Directed Mutagenesis Kit, Agilent 200521) using the following primers: shARG2-A10-resistant-F (5′-ACACGTGCTTGATTTTCGGATTCGTCTGGGGAACTGGGAGTAGGAAG-3′) and shARG2-A10-resistant-R (5′-CTTCCTACTCCCAGTTCCCCAGACGAATCCGAAAATCAAGCACGTGT-3′). Retroviral and lentiviral supernatants were generated by transfecting the above constructs into 293T cells and used to infect AsPC-1 cells. The infected cells were selected for at least 7 days 1200 μg ml^−1^ G418 (Sigma-Aldrich, A1720).

### Lentiviral-mediated shRNA targets

The RNAi consortium clone IDs for the shRNAs used in this study are as follows: shARG2-A06 TRCN0000051018 (CGAACATTTGATCTGCTGATT); shARG2-A07 TRCN0000051019 (CCTATCGAGAAGGCATGTATA); shARG2-A10 TRCN0000051022 (GTTCACCAGATGAATCAGAAA); shARG2-B09 TRCN0000369866 (GAACTATGATATCCAGTATTT); shARG2-B10 TRCN0000333446 (CCCTTACCACTTCATCAGGAA); shGFP TRCN0000072182 (TCTCGGCATGGACGAGCTGTA). Scramble shRNA (CCTAAGGTTAAGTCGCCCTCGCTCGAGCGAGGGCGACTTAACCTTAGG) was from Addgene (Plasmid # 1864). For generation of PDA cells with stable *ARG2* or control (Scramble or GFP) knockdown, lentiviral supernatants produced from pLKO plasmids encoding the corresponding hairpins were used, and infected cells were selected for at least 7 days with 4 μg ml^−1^ puromycin (Sigma-Aldrich, P8833).

### Animal work

All animal studies and procedures were approved by the Animal Care and Use Committee at Boston Children’s Hospital. No statistical methods were used to predetermine sample size. For orthotopic xenografts, 10^5^ to 10^6^ cells suspended in 25 μl 33% Matrigel (BD Biosciences 356231) in HBSS were injected into the pancreata of lean or obese male B6.129S7-*Rag1* ^*tm1Mom*^/J mice termed *Rag1* ^−/−^ mice (Jackson Laboratory #002216) at least 4 weeks after special diet feeding. KPC-*Arg2* ^*+/+*^ and KPC-*Arg2* ^−/−^ mice were generated by crossing KPC mice or *LSL-Kras* ^*G12D*^ *;p53* ^*fl/fl*^ *mice;pdx-1-Cre* ^42, 43^ (C57BL/6J-129 svJae) to *Arg2* ^*tm1Weo*^ */J* or *Arg2* ^−/−^ mice (C57BL/6J, Jackson Laboratory #020286). For immune-proficient orthotopic transplants models, 250,000–500,000 mouse PDA cells derived from KPC tumors were orthotopically injected into littermates harboring wild-type *Kras* and *Arg2* alleles. The experiments were not randomized. The investigators were not blinded to allocation during experiments or outcome assessment.

### Diet feeding and plasma analysis

4–12 week old mice were fed ad libitum either a control chow diet (Harlan Teklad #7001, 3 Kcal g^−1^, 13% Kcal from fat) or a high fat diet (D12492, Research Diets, 5.24 Kcal g^−1^, 60% Kcal from fat) and monitored for several weeks. Body weight and food intake were measured and recorded weekly. Glucose tolerance test after a 24-h fast was performed as previously described^44^. Plasma glucose levels were measured using a glucose oxidase kit (Thermo Fisher Scientific). Plasma insulin and IGF-I were assayed using kits from CrystalChem and Diagnostic Systems Laboratories, respectively.

### Necropsy

Mice were euthanized at the beginning of the light cycle after retro-orbital blood withdrawal, and plasma was prepared as previously described^44^. Tumors were collected, their dimensions (termed *a*, *b* and *c*) were measured with a caliper and tumor volume was estimated according to the ellipsoid formula^45^: 4/3 × π × (*a*/2 × *b*/2 × *c*/2). Tumors were then either immediately frozen in liquid nitrogen, or fixed in formalin for later processing.

### Immunoblotting

Cells were rinsed once in ice-cold PBS and collected in lysis buffer containing 50 mM HEPES, pH 7.4, 40 mM NaCl, 2 mM EDTA, 1.5 mM orthovanadate, 50 mM NaF, 10 mM pyrophosphate, 10 mM glycerophosphate, EDTA-free protease inhibitors (Roche) and 1% Triton X-100. Tumor tissues were homogenized in lysis buffer supplemented with 1% deoxycholate and 0.1% SDS. Proteins from total lysates were resolved by 8–12% SDS–PAGE, proteins were transferred to polyvinylidene difluoride and the blot was exposed to film. In blots assessing ARG1 and ARG2 levels, protein lysates from human hepatocellular carcinoma HepG2 cells were used as a positive control. For all blots, glyceraldehyde dehydrogenase (GAPDH) was used as a loading control. Protein band intensity was quantified using Image J software (NIH). Scanned films of uncropped blots are shown in Supplementary Fig. 11.

### Immunohistochemistry and analysis of human samples

Formalin-fixed paraffin-embedded tumor samples were sectioned and immunostained according to the manufacturers’ protocols. For human samples, a pathologist scored, in a blinded fashion, the intensity of ARG2 and pS473-AKT staining in PDA cases on tissue microarrays with 2–7 cores per case including non-neoplastic pancreatic tissue as control. For ARG2 staining, the intensity was scored on a scale of 0–3 that represented none (0), weak (1), moderate (2), and strong (3) based on the staining seen in the majority of lesional cells. For pS473-AKT staining, scores for each tumor core were estimated using the following formula: intensity score = 0 × (% area of tumor with no staining) + 1 × (% tumor area with moderate staining) + 2 × (% tumor area with strong staining), where 200 would be the highest score. Analysis of human subjects included PDA tissue microarrays (TMAs) from 92 non-diabetic patients with surgically resected PDA (58 from Massachusetts General Hospital or MGH and 34 from Dana-Farber/Brigham and Women’s Cancer Center or DF/BWCC). For MGH patients, informed consent was waived by the Institutional Review Board or IRB (Protocol Number 2009P001838), as the TMAs were generated from discarded material following clinical diagnosis. For DF/BWCC patients, informed consent for use of clinical data and tissue specimens was obtained with approval by the Dana-Farber/Harvard Cancer Center or DF/HCC IRB (Protocol Numbers 03-189 and 02-023). Patient height and weight were obtained from preoperative evaluation within 2 weeks before surgery and used to calculate BMI, which was modeled as a continuous variable. pAKT and ARG2 were evaluated as dichotomous categorical variables. Levels of each protein were classified as “high” if ≥ 25th percentile stain score among the 92 samples. Analyses of the associations between BMI, pAKT, and ARG2 levels were conducted using binomial logistic regression, obtaining odds ratios (ORs) and 95% confidence intervals (95% CI). The ORs reflect the odds per unit increase in BMI (kg m^−2^) for a high ARG2 expressing tumor, or the odds of high versus low-pAKT levels for a high-ARG2 expressing tumor. Regression models were adjusted for age at surgery, sex, and American Joint Committee on Cancer (AJCC) 7th edition pathological stage (IA-IIA, IIB-III). All hypothesis tests were two-sided. Statistical significance was set at *α* = 0.05. Analyses were performed using SAS software (version 9.4, SAS Institute, Cary, NC).

### Gene expression profiling and GSEA

Tumor tissues were homogenized and control AsPC1-CFP cultured cells were lysed in QIAzol lysis reagent and total RNA was extracted using the RNeasy Microarray Tissue Mini Kit (Qiagen). RNA quality was checked using the Bioanalyzer RNA Nano kit, and 325 ng were used for microarray labeling with the Agilent LowInput QuickAmp Labeling Kit Two-Color. Dye incorporation and yield were measured with a Nanodrop spectrophotometer. Equal amounts of differentially labeled control AsPC1-CFP cells propagated in culture and sample cRNA were combined such that each sample contained at least 2.5 pmol dye. Samples were mixed with control targets, fragmented, combined with hybridization buffer, and hybridized to the SurePrint G3 Human Gene Expression 8 × 60 K v2 Microarray consisting of 60mer probes for each human open reading frame (Agilent). Microarrays were rotated at 60 °C for 17 h in a hybridization oven (Agilent). Arrays were then washed according to the Agilent SSPE wash protocol, and scanned on an Agilent scanner. The image was processed using the default settings with Agilent Feature Extraction software. All data analysis was performed using the resulting log(2) ratio data of tumor samples over AsPC1-CFP cell line control samples, and filtered for spots called as significantly over background in at least one channel. For GSEA^22, 46^, gene sets collection from Kyoto Encyclopedia of Genes and Genomes (KEGG) were included in the analysis to assess the enrichment of the obese and AKT gene signatures. A pairwise GSEA was performed by creating ranked lists of genes using the difference of class means to calculate fold change for log scale data between the Lean-CFP and Obese-CFP (Fig. 1d) or between Lean-CFP and the rest of the tumors grouped together (Obese-CFP, Lean-AKT and Obese-AKT, Fig. 4f) and *P* values were obtained from permuting the gene set (1000 permutations).

### Metabolite extraction and quantification

Tissues (10–30 mg) were homogenized using a Qiagen TissueLyzer II in 1:4 (w:v) water. 10 µl of each tissue homogenate was combined with 90 µl of extraction solution (acetonitrile:methanol:formic acid (75:25,0.2v:v:v), 0.2 ng µl^−1^ d8-phenylalanine and d8-valine), vortexed, centrifuged (10 min, 10,000 × *g*, 4 °C) and the supernatants collected and stored (−80 °C) for metabolite profiling. Data were acquired using a hydrophilic interaction liquid chromatography method (HILIC) with positive ion mode Mass Spectrometry (MS) operated on Nexera X2 UHPLC (Shimadzu Scientific Instruments, Marlborough, MA) coupled to a Q Exactive orbitrap mass spectrometer (Thermo Fisher Scientific, Waltham, MA) as described previously^47^, with data collected in the 70–800 *m*/*z* range. For experiments measuring urea, data were collected in the 55–750 *m*/*z* range. The supernatants (10 µl) were injected directly onto a 150 × 2 mm Atlantis HILIC column (Waters; Milford, MA). The column was eluted isocratically at a flow rate of 250 μl min^−1^ with 5% mobile phase A (10 mM ammonium formate and 0.1% formic acid in water) for 1 min followed by a linear gradient to 40% mobile phase B (acetonitrile with 0.1% formic acid) over 10 min. The electrospray ionization voltage was 3.5 kV. LC–MS data were processed and visually inspected using TraceFinder 3.3 software. For global steady-state metabolite profiling, data were generated by Metaboanalyst 3.0^48^, median-normalized, log-transformed, mean-centered and divided by the s.d. of each variable. In addition to LC–MS, urea levels were quantified using a urea assay kit (Sigma #MAK006). For metabolic tracing in cultured cells, dried extracts were suspended in 100 µl water. After centrifugation at top speed for 10 min, 2 µl of supernatant was injected for LC/MS analysis as described in ref. ^49^.

### Ammonia derivatization

In total 10 μl of 1 mM ethylamine internal standard (in water) was directly added to tissue chunks, followed by addition of 250 μl of 0.5 mg ml^−1^ dansyl chloride (in acetonitrile) and homogenization on ice. The homogenates were centrifuged at top speed at 4 °C for 5 min, the supernatants collected and incubated at 70 °C for 5 h, followed by centrifugation at top speed at 4 °C for 5 min. The supernatants were then stored at −80 °C. In total 1 μl of supernatant was injected for LC–MS analysis. Chromatographic separation was performed on a Dionex Ultimate 3000 UPLC system using a Kinetex 50 × 2.1 mm C18 (100 Å, 2.6 μm particle size) column (Phenomenex). The mobile phases were 0.1% formic acid in water (mobile phase A) and 0.1% formic acid in acetonitrile (mobile phase B). The flow rate was held at 0.4 ml min^−1^ and the column compartment was held at 35 °C. The chromatographic gradient was as follows: 0–3.5 min.: linear increase from 5% B to 80% B; 3.5–3.6 min: linear increase from 80% B to 98% B; 3.6–4.5 min.: hold at 98% B; 4.5–4.6 min.: linear decrease from 98% B to 5% B; 4.6–6.0 min.: hold at 5% B. The column eluate was introduced directly into the ionization source of a QExactive benchtop orbitrap mass spectrometer (Thermo Fisher Scientific) equipped with a HESI probe. The mass spectrometer was operated in positive ionization mode, using targeted selected ion monitoring (tSIM) scans with target windows of width 2.0 *m*/*z* centered at *m*/*z* = 251.5834 (to include dansylated ammonia and 15N-ammonia) and *m*/*z* = 279.1162 (to include dansylated ethylamine). The resolution was set to 140,000, the AGC target was set to 1e5, and the maximum integration time was set to 250 ms. The spray voltage was set to 3.0 kV, the capillary temperature was set to 275 °C, the sheath gas was set to 40 units, the auxiliary gas was set to 15 units, and the spare gas was set to 1 unit. The probe heater temperature was set to 350 °C and the S-lens RF level set to 40 units. External mass calibration was performed using the standard calibration mixture every 7 days. Data were analyzed using XCalibur QuanBrowser v2.2 (Thermo Fisher Scientific). The chromatographic retention times of the derivatization products were confirmed by analyzing a derivatization reaction containing pure ammonia and ethylamine. The relative abundance of ^15^N-ammonia (Fig. 3h) is reported as the raw peak area of dansylated ^15^N-ammonia divided by the raw peak area of dansylated ethylamine, whereas the fraction labeled is reported as the raw peak area of dansylated 15N-ammonia divided by the summed raw peak areas of dansylated ^15^N- and unlabeled ammonia.

### Infusion of labeled nutrients

Infusions were performed as previously described in ref. ^38^. Specifically, lean or obese immunodeficient mice bearing human orthotopic pancreatic xenografts were infused 3 weeks after the injection of tumor cells, with ^15^N(amine)-glutamine (99% enrichment; Cambridge Isotope Laboratories, Andover, MA) as a bolus of 0.28 mg g^−1^ body weight (0.3 ml) administered over 1 min, followed by a continuous infusion of 0.005 mg g^−1^ min^−1^ for 180 min. Alternatively, immune-proficient lean or obese mice bearing mouse orthotopic transplant tumors were infused 2 weeks after the injection of tumor cells, with ^13^C_6_,^15^N_4_-arginine.HCl (^13^C6, 99%; ^15^N4, 99% enrichment, Cambridge Isotope Laboratories, Andover, MA) as a bolus of 0.084 mg g^−1^ body weight (0.3 ml) over 1 min, followed by a continuous infusion of 0.0015 mg g^−1^ min^−1^ for 180 min. In total 25 μl of tail blood was collected at 30 min intervals and used to quantify plasma enrichment of labeled nutrients by LC–MS. At the end of the infusions, mice were euthanized and the tumors rapidly harvested, weighed and snap-frozen in liquid nitrogen for LC–MS analysis.

### Statistics

Statistical analyses are expressed as mean ± s.d., or s.e.m., unless otherwise indicated. No statistical methods were used to predetermine sample size. In comparing two groups, a two-tailed non-paired Student *t*-test was conducted. For three or more groups, one-way ANOVA was conducted, except for growth curves (two-way ANOVA), followed by a post hoc Tukey test. *P* ≤ 0.05 was considered statistically significant. For GSEA and metabolic pathway analysis, a FDR *q*-value ≤ 0.25 was considered significant.

### Data availability

The microarray data have been deposited in NCBI’s Gene Expression Omnibus^50^ and are accessible through GEO Series accession number GSE99289. The authors declare that all the other data supporting the findings of this study are available within the article, its Supplementary Information and from the corresponding author upon reasonable request.

## Electronic supplementary material

Supplementary Information
