# Supplementary material for: Critical role for arginase 2 in obesity-associated pancreatic cancer
Source: Nat Commun. 2017 Aug 14;8:242. doi: 10.1038/s41467-017-00331-y (PMC5556090; doi:10.1038/s41467-017-00331-y)
Supplement: Supplementary file 1 — Supplementary Information [file 41467_2017_331_MOESM1_ESM.pdf]

File Name: Supplementary Information
Description: Supplementary Figures

a

Control Chow	%
Protein	25.2
Fat	4.4
Carbohydrates	39.5
Calories from Protein	34
Calories from Fat	13
Calories from Carbohydrates	53

HFD	%
Protein	26.2
Fat	34.9
Carbohydrates	26.3
Calories from Protein	20
Calories from Fat	60
Calories from Carbohydrates	20

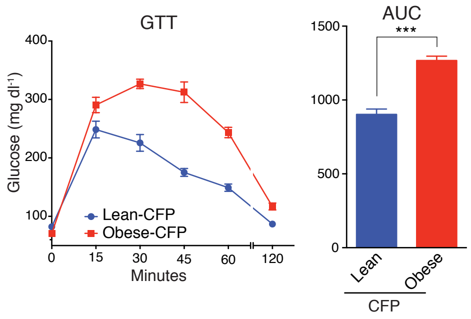

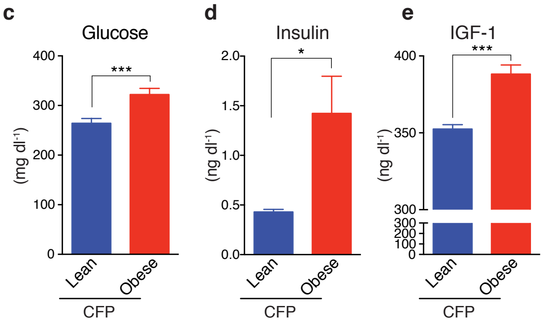

Supplementary Figure 1. Obese mice bearing AsPC1-CFP tumours display features of the metabolic syndrome. (**a**) Table describing the composition of the control chow and high fat diet (HFD) fed to mice in the current study. (**b**) Glucose tolerance test (GTT) performed on mice (n = 15 lean and n = 14 obese) from **Figure 1b**, 7 weeks post-HFD feeding (left) and quantification of the area under the curve (AUC, right). (**c-e**) Plasma glucose (**c**) insulin (**d**) and total IGF-1 (**e**) levels in mice from **Figure 1b**, at time of euthanasia (n = 15). Data represent the mean ± s.e.m. in **b**, and mean ± s.d. in **c-e**. *$P \leq 0.05$, ***$P \leq 0.001$ by *t*-test.

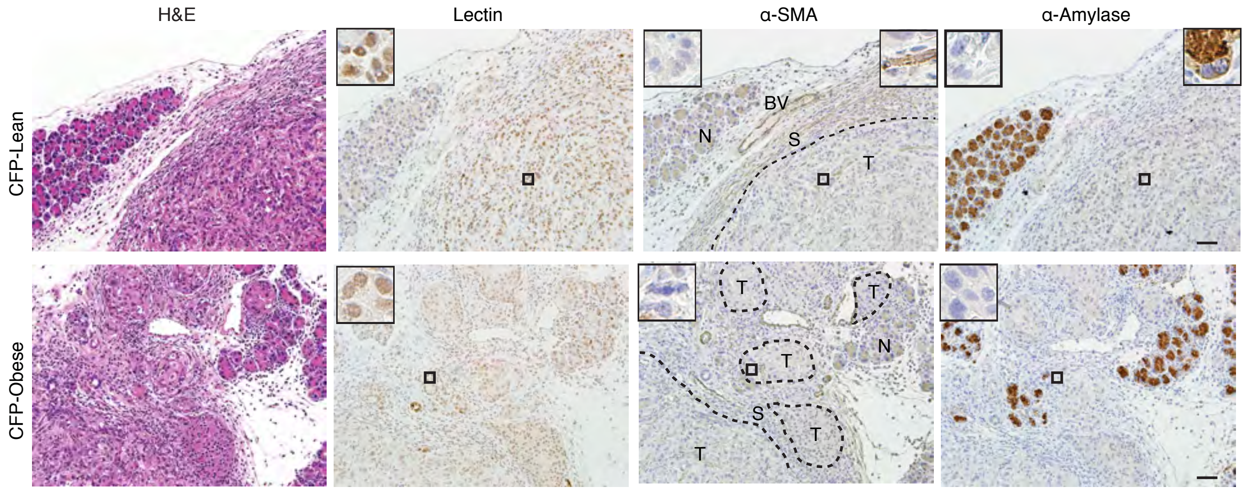

Supplementary Figure 2. **Human PDA tumours display mild stromal infiltration in both lean and obese mice.** Haematoxylin and Eosin stain (H&E, left) and immunohistochemistry of DBA-Lectin, α-Smooth Muscle Actin (α-SMA) and α-Amylase, which stain respectively, ductal cells (normal and neoplastic), stromal cells, and acinar cells, in representative human AsCP1-CFP PDA tumours described in **Figure 1c**. Dashed lines demarcate areas of ductal adenocarcinoma; T, tumour; N, normal acinar tissue; S, stroma; BV, blood vessel. Framed top corner insets represent a 5-fold magnification of the area indicated by a square in the tumour (left), or a 5-fold magnification of stain-positivity for either α-SMA or α-Amylase (right). Scale bars: 50 μm.

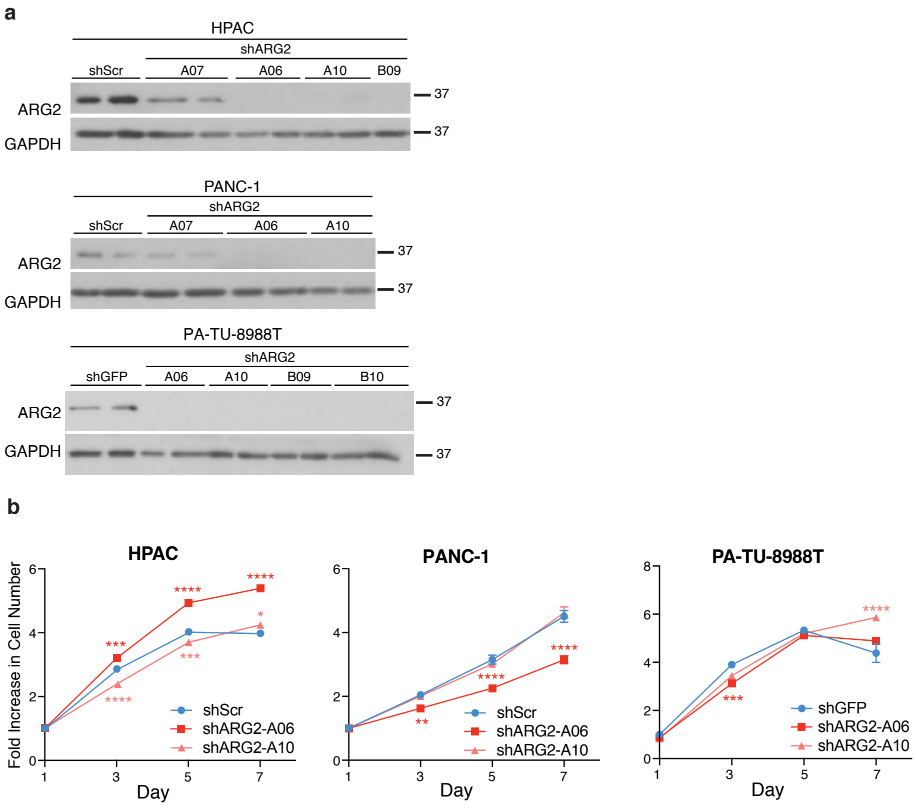

Supplementary Figure 3. ARG2 knockdown does not suppress *in vitro* PDA cell growth. (**a**) Immunoblots of ARG2 in HPAC, PANC-1 and PA-TU-8988T PDA cells showing decreased levels upon ARG2 knockdown (hairpins A06, A07, A10, B09 and B10), compared to control knockdown (shScr or shGFP). (**b**) Proliferation curves of cells in **a** demonstrating that ARG2 silencing does not significantly decrease the growth of cultured PDA cells (n = 6). Data are representative of 2 independent experiments and represent the mean ± s.e.m. *$P \leq 0.05$, **$P \leq 0.01$, ***$P \leq 0.001$, ****$P \leq 0.0001$ for differences between control cells and cells with ARG2 knockdown at the indicated timepoints as assessed by two-way ANOVA followed by Tukey test.

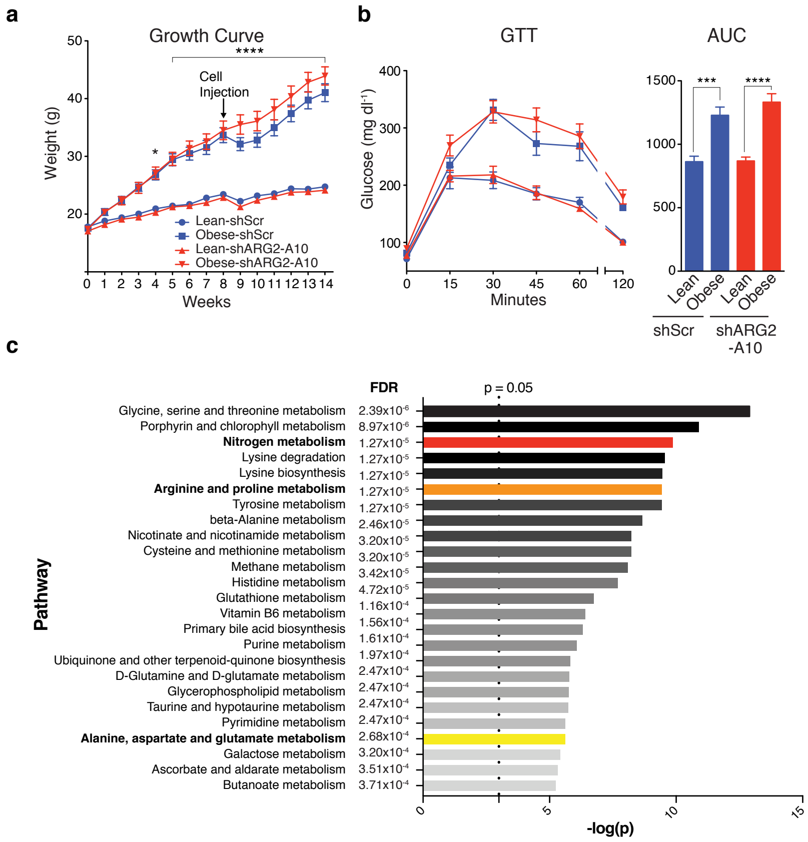

Supplementary Figure 4. Nitrogen metabolism pathways are enriched upon *ARG2* knockdown in human PDA tumours. (**a**) Growth curves for 4-6 week old male *Rag1*$^{-/-}$ mice described in **Figure 3a** that were fed either chow or HFD (n = 7 for shScr and n = 8 for shARG2) for 14 weeks. Arrow indicates orthotopic injection of control (AsPC1-shScr) or *ARG2* knockdown (AsPC1-shARG2-A10) cells into the pancreata of lean or obese mice. (**b**) Glucose tolerance test (GTT) performed on mice (n = 7-8) in **a**, 7 weeks post-HFD feeding (left) and quantification of the area under the curve in (AUC, right). (**c**) List of top metabolic pathways that are differentially altered upon *ARG2* knockdown in AsPC-1 PDA tumours described in **Figure 3a**. Metabolite data were processed by Metaboanalyst 3.0 and the pathways were ranked by –log(2) of the *P*-value. In color are pathways involved in nitrogen and arginine metabolism that are enriched in urea cycle metabolites. In **a**,**b**, data represent the mean ± s.e.m. $*P \leq 0.05$, $***P \leq 0.001$, $****P \leq 0.0001$, by two-way ANOVA in **a**, and one-way ANOVA in **b**, followed by Tukey test.

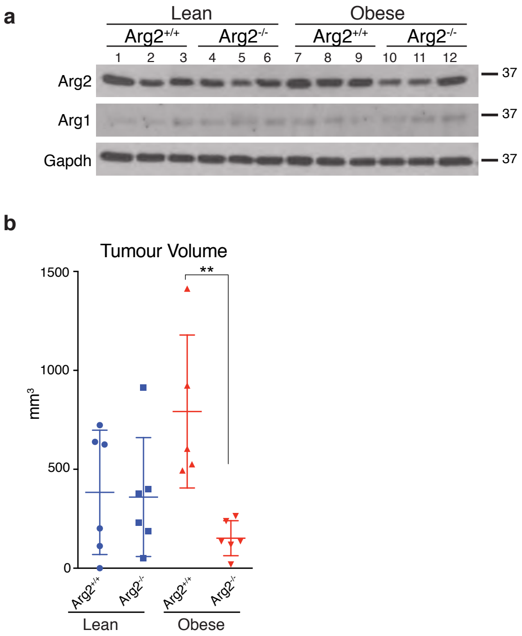

Supplementary Figure 5. ***Arg2*** **loss suppresses the growth of KPC tumours in obese mice.** (**a**) Immunoblots for Arg2 and Arg1 from tumours described in **Figure 5c**, showing that aberrant, inactive Arg2 is detected by western blotting in mouse KPC; *Arg2*$^{-/-}$ tumours. (**b**) Volumes of orthotopic PDA tumours derived from mouse KPC cells with or without *Arg2* loss, that were injected into the pancreata of female lean or obese 16-18 week old C57BL/6J-129 svJae mice (500,000 cells per mouse) and grown for 3 weeks; n = 6 from 2 independent experiments. Data represent mean ± s.d. $^{**}P \leq 0.01$ by one-way ANOVA followed by Tukey test.

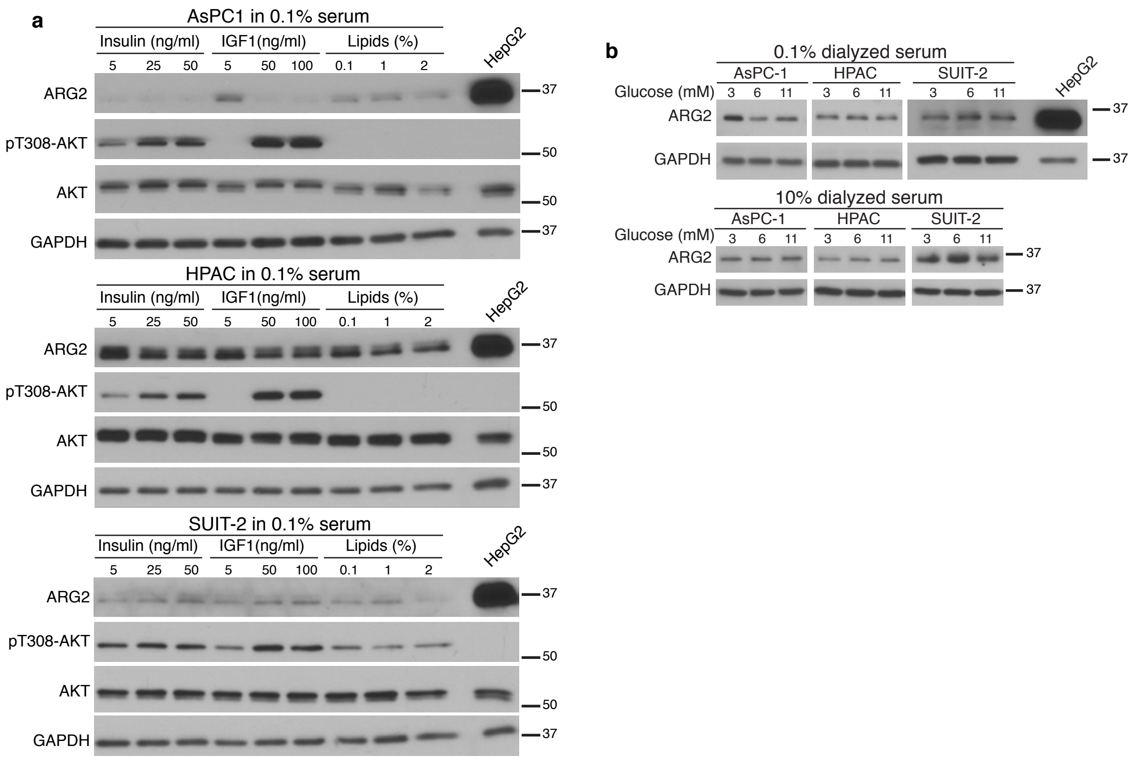

Supplementary Figure 6. ARG2 expression is not induced *in vitro* by insulin, IGF-1, glucose or lipids. (**a**) Immunoblots of ARG2, pT308-AKT and total AKT in AsPC1, HPAC and SUIT-2 human PDA cells grown in 0.1% serum and treated with the indicated levels of insulin, IGF-1 or lipid mixture for 24 hours. (**b**) Immunoblots of ARG2 in cells described in **a** that were grown in 0.1% (top) or 10% (bottom) dialyzed serum and treated with the indicated levels of glucose for 24 hours. Data in **a**,**b** are representative of 2 independent experiments.

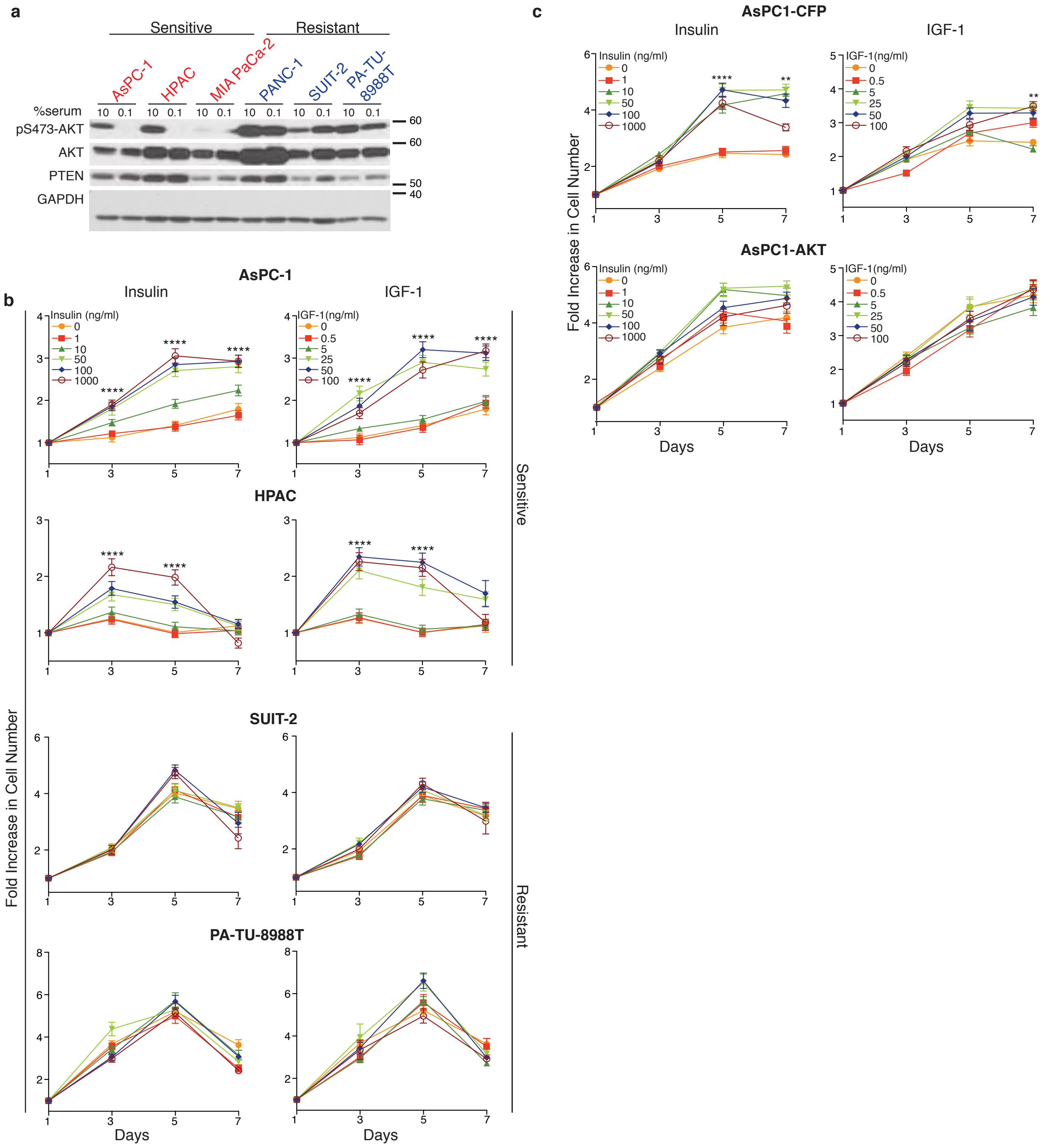

Supplementary Figure 7. Differential AKT activation and sensitivity to growth factors in human PDA cells. (**a**) Immunoblots for pS473-AKT, total AKT and PTEN in human PDA cells grown in 10% or 0.1% serum, indicating constitutive AKT activation in "serum-resistant" compared to "serum-sensitive" cells. (**b**) Proliferation curves of PDA cells described in **a** showing differential sensitivity to insulin or IGF-1, of "serum-sensitive" cells, compared to growth factor-independent "serum-resistant" cells. All cells were cultured in the presence of 0.1% serum and increasing concentrations of insulin or IGF-1 (n = 6). Data are representative of 2 independent experiments. (**c**) Proliferation curves of AsPC1-AKT and AsPC1-CFP cells cultured as in **b**, showing differential sensitivity of AsPC1-CFP, but not AsPC1-AKT cells to insulin or IGF-1 (n = 6). For all graphs, data represent the mean ± s.e.m. $^{**}P \leq 0.01$, $^{****}P \leq 0.0001$ for differences between 0 and 1000 ng/ml^{-1} insulin, or between 0 and 100 ng ml^{-1} IGF-1 at the indicated timepoints. Two-way ANOVA was conducted followed by Tukey test.

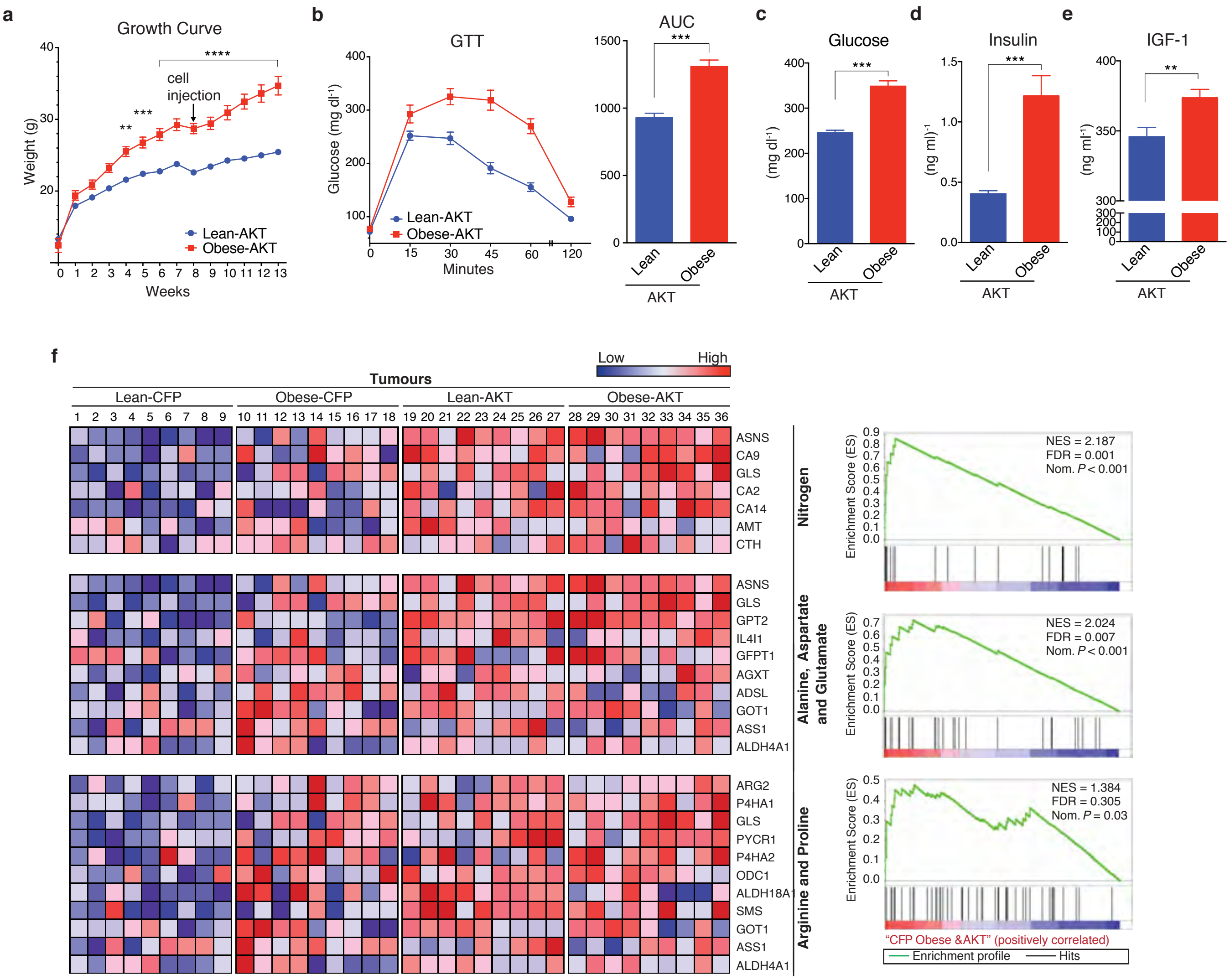

Supplementary Figure 8. Activated AKT leads to transcriptional induction of nitrogen and arginine metabolic pathways in PDA tumours independent of obesity. (**a**) Growth curves of 4-6 week old male *Rag1*$^{-/-}$ mice described in **Figure 6b** that were fed either chow (Lean-AKT, n =14) or HFD (Obese-AKT, n =15). Arrow indicates orthotopic injection of AsPC1-CFP or AsPC1-AKT cells (10^5 per mouse) into the pancreata of lean or obese mice. (**b**) Glucose tolerance test (GTT) performed on mice (n = 14 for Lean-AKT and n = 15 for Obese-AKT) described in **a**, 7 weeks post-HFD feeding (left) and quantification of the area under the curve (AUC, right). **c-e**, Plasma glucose (**c**), insulin (**d**) and total IGF-1 (**e**) levels in Lean-AKT and Obese-AKT mice, at time of euthanasia (n = 14 for lean and n = 15 for obese). (**f**) Heatmaps (left) of the induced genes in indicated metabolic pathways with corresponding enrichment plots (right), illustrating differential gene up-regulation in AsPC1-CFP tumours grown in obese mice (Obese-CFP, 10-18) and all AsPC1-AKT tumours (Lean-AKT, 19-27; Obese-AKT: 28-36), compared to AsPC1-CFP tumours grown in lean mice (Lean-CFP, 1-9). Red signal indicates higher expression, and blue indicates lower expression relative to the mean expression level within each group. NES, normalized enrichment scores; FDR false discovery rate; Nom., nominal. In **a**,**b**, data represent the mean ± s.e.m. In **c-e**, data represent the mean ± s.d. **$P \leq 0.001$, ***$P \leq 0.001$, ****$P \leq 0.0001$, by two-way ANOVA followed by Tukey test in **a**, and by *t*-test in **b-e**.

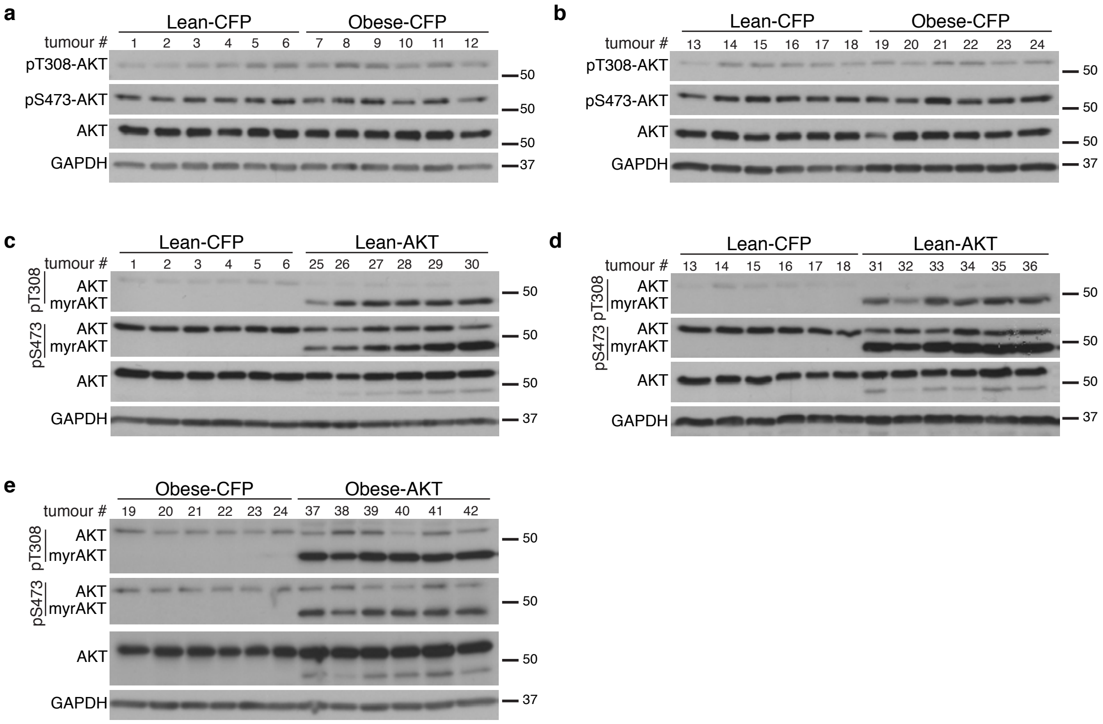

Supplementary Figure 9. AKT phosphorylation is not increased in PDA tumours of the obese. (**a-d**) Immunoblots for pT308-AKT, pS473-AKT and total AKT in AsPC1-CFP and AsPC1-AKT PDA tumours grown in lean or obese mice described in **Figures 1c** and **6b**.

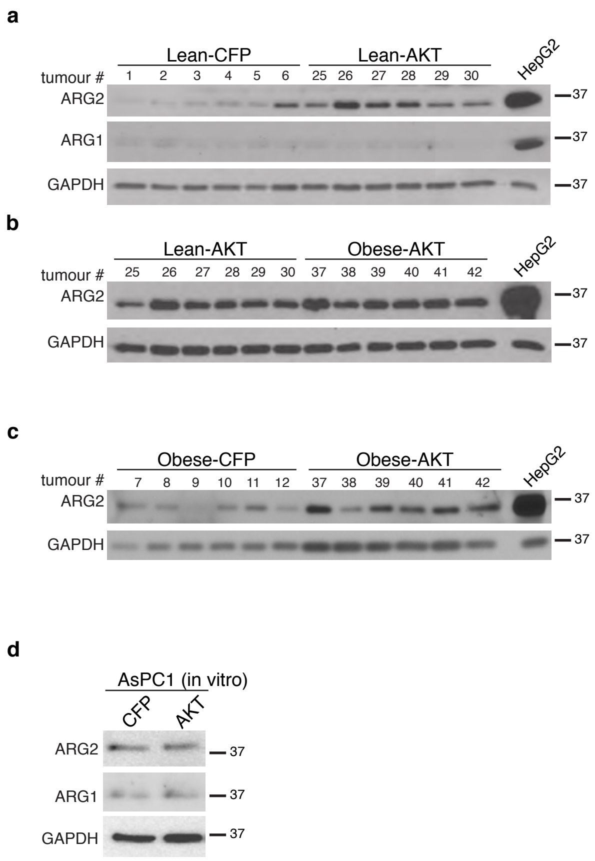

Supplementary Figure 10. ARG2 is induced in obesity-associated and AKT-driven PDA *in vivo* but not *in vitro*. (**a-c**) Immunoblots of ARG2 and ARG1 in orthotopic AsPC1-CFP and AsPC1-AKT tumours described in **Figures 1c** and **6b**, showing increased ARG2 levels in all AKT-driven tumours, independent of obesity. (**d**) Immunoblots of ARG2 and ARG1 in cultured AsPC1-CFP and AsPC1-AKT cells, showing lack of induction of ARG2 expression by activated AKT *in vitro*.

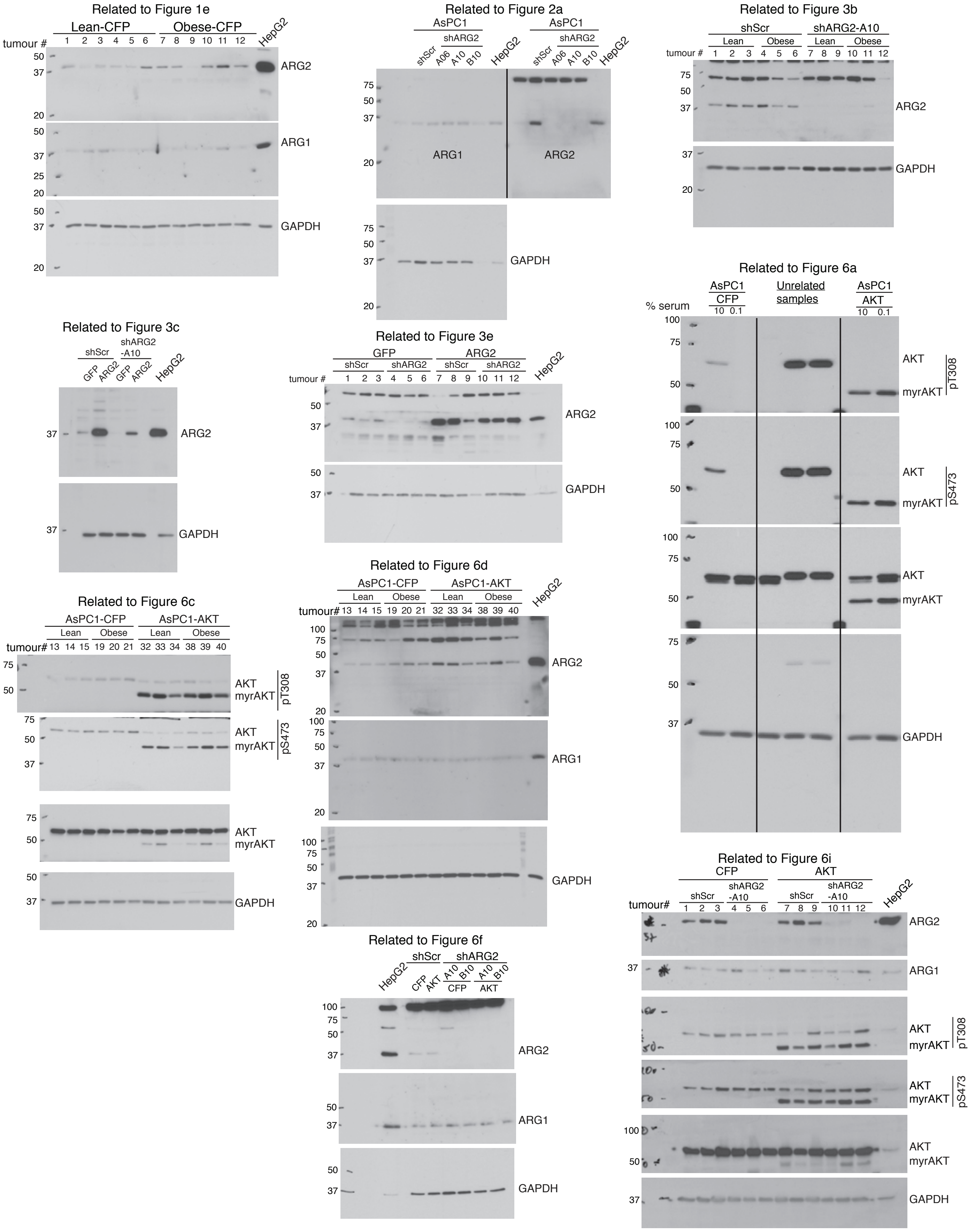

Supplementary Figure 11. Scanned films of uncropped Western blots related to the indicated panels from Main Figures 1, 2, 3 and 6. Blots for different antibodies that were developed on the same film (i.e. **Figure 2a**) or blots including unrelated samples (i.e. **Figure 6a**) are separated by a black line.
